# Identification of Serum Biomarkers for Biliary Tract Cancers by a Proteomic Approach Based on Time-of-Flight Mass Spectrometry

**DOI:** 10.3390/cancers2031602

**Published:** 2010-08-18

**Authors:** Wen-Jing Wang, Wang-Hong Xu, Cha-Zhen Liu, Asif Rashid, Jia-Rong Cheng, Ping Liao, Heng Hu, Lisa W. Chu, Yu-Tang Gao, Kai Yu, Ann W. Hsing

**Affiliations:** 1 Department of Molecular Biology for Public Health, Shanghai Municipal Center for Disease Control and Prevention, 1380 Zhong Shan Xi Road, Shanghai, 200336, China; E-Mails: wjwang@scdc.sh.cn (W.J.W.); czliu@scdc.sh.cn (C.Z.L); pliao@scdc.sh.cn (P.L.); pliao@scdc.sh.cn (H.H.); 2 Department of Epidemiology, Shanghai Cancer Institute, 2200/25 Xie Tu Road, Shanghai, 200032, China; E-Mails: jrcheng@yahoo.com (J.R.C); ytgao@vip.sina.com (Y.T.G); 3 Department of Epidemiology, School of Public Health; Key Laboratory of Public Health Safety, Ministry of Education, Fudan University,138 Yi Xue Yuan Road, Shanghai, 200032, China;E-Mail: wanghong.xu@fudan.edu.cn (W.H.X.); 4 Department of Pathology, MD Anderson Cancer Center, Houston, TX, USA; E-Mail: arashid@mail.mdanderson.org (A.R.); 5 Division of Cancer Epidemiology and Genetics, Department of Health and Human Services, National Cancer Institute, Bethesda, MD 20892-7234, USA; E-Mails: chulisa@mail.nih.gov (L.W.C.); yuka@mail.nih.gov (K.Y.); hsinga@mail.nih.gov (A.W.H.)

**Keywords:** biliary tract cancers (BTCs), surface-enhanced laser desorption/ionization mass spectrometry (SELDI-TOF-MS), tumor biomarker, protein profiling

## Abstract

Biliary tract cancers (BTCs) are lethal malignancies currently lacking satisfactory methods for early detection and accurate diagnosis. Surface-enhanced laser desorption/ionization time-of-flight mass spectrometry (SELDI-TOF-MS) is a promising diagnostic tool for this disease. In this pilot study, sera samples from 50 BTCs and 30 cholelithiasis patients as well as 30 healthy subjects from a population-based case-control study were randomly grouped into training set (30 BTCs, 20 cholelithiasis and 20 controls), duplicate of training set, and blind set (20 BTCs, 10 cholelithiasis and 10 controls); all sets were analyzed on Immobilized Metal Affinity Capture ProteinChips via SELDI-TOF-MS. A decision tree classifier was built using the training set and applied to all test sets. The classification tree constructed with the 3,400, 4,502, 5,680, 7,598, and 11,242 mass-to-charge ratio (*m/z*) protein peaks had a sensitivity of 96.7% and a specificity of 85.0% when comparing BTCs with non-cancers. When applied to the duplicate set, sensitivity was 66.7% and specificity was 70.0%, while in the blind set, sensitivity was 95.0% and specificity was 75.0%. Positive predictive values of the training, duplicate, and blind sets were 82.9%, 62.5% and 79.2%, respectively. The agreement of the training and duplicate sets was 71.4% (Kappa = 0.43, u = 3.98, P < 0.01). The coefficient of variations based on 10 replicates of one sample for the five differential peaks were 15.8–68.8% for intensity and 0–0.05% for m/z. These pilot results suggest that serum protein profiling by SELDI-TOF-MS may be a promising approach for identifying BTCs but low assay reproducibility may limit its application in clinical practice.

## 1. Introduction

Biliary tract cancers (BTCs), encompassing tumors of gallbladder, extrahepatic bile ducts, and ampulla of Vater, are rare but fatal malignancies. During the past several decades, incidence of BTCs has increased rapidly in Shanghai, China, with the age-adjusted incidence of BTCs in males and females going from 1.2 to 3.1 per 100,000 and from 1.7 to 4.6 per 100,000 between 1972–1974 and 1993–1994, respectively; the annual percentage change was 4.0% and total percentage change was 160% [1,2]. 

Due to the difficult accessibility of diagnostic tissues and lack of specific symptoms, most BTCs are often detected at an advanced stage, resulting in average 5-year relative survival rates of 10–20% [3]. However, survival rates are closely related with stage at diagnosis. In one study, the 5-year survival rates were only 0.7% for gallbladder cancer and 1.3% for extrahepatic bile duct cancer with metastasis, compared to 41.9% and 26.5% for localized cancers, respectively [3]. Donohue *et al.* [4] also reported an after-surgery 5-year survival rate of less than 5% for gallbladder cancer in patients diagnosed at a later stage, but an 85%–91% rate for those diagnosed at an early stage. In a Chinese population, the survival rate of gallbladder cancer was also closely associated with the stage at diagnosis [5]. Thus methods that could aid in early detection are desirable for better outcomes.

Differentiation of benign and malignant lesions is also important for the prognosis of the disease. Currently, BTCs are usually diagnosed through a combination of imaging, endoscopy histology and blood tests. The imaging and endoscopy techniques, such as magnetic resonance imaging (MRI), computed tomography (CT), endoscopic retrograde cholangiopancreatography (ERCP) and magnetic resonance cholangiopancreatography (MRCP), perform well in locating the lesions but lack specificity in distinguishing cancers from inflammatory or non-neoplasitc entities. For example, gallbladder cancer and cholecystitis cannot be reliably distinguished by the currently available diagnostic methods [6]. In the Shanghai Biliary Tract Cancer study (SBTCS), the parent study of this report, about 21% of the cancers were incidental findings through cholecystectomy; the misdiagnosis rates of extrahepatic bile ducts cancer and ampulla of Vater cancer reached 19.1% and 47.1%, respectively [5]. Serum levels of carcinoembryonic antigen (CEA) and CA19-9 are often elevated in BTCs, but are not sensitive or specific enough to be used for differential diagnosis of BTCs [7,8]; instead they are used in conjunction with imaging techniques but still have low sensitivity and specificity [9]. Due to these limitations, the diagnosis of BTCs sometimes has to include more invasive surgical exploration. More non-invasive methods that could discriminate BTCs from non-cancers are needed.

Serum assays using surface-enhanced laser desorption/ionization time-of-flight mass spectrometry (SELDI-TOF-MS), a high throughput technique to detect protein expression profiles, is a relatively non-invasive method that has been used to detect differentially expressed proteins in a series of cancers [10,11,12,13], including intrahepatic cholangiocarcinoma [14,15]. In this study, we tested the application of using SELDI-TOF-MS to identify novel serum protein profiles that are differentially expressed in BTCs compared to controls in the SBTCS [16].

## 2. Material and Methods

### 2.1. Subjects and Samples

Fifty BTCs, 30 cholelithiasis and 30 healthy controls were randomly selected from the participants of the SBTCS, a population-based case-control study conducted by the Shanghai Cancer Institute and the National Cancer Institute between 1997 and 2001. As described in our previous reports [16,17,18,19,20,21,22], the BTCs were identified through a rapid reporting system and confirmed by a panel of clinicians, ultrasongraphers and pathologists through reviewing pathologic slides, CT, MRI and ERCP and medical records in a blind fashion. The blood samples of BTCs were collected after diagnosis but before treatment, and blood samples of cholelithiasis subjects and controls were drawn at interview. All samples were processed within four hours of collection and stored at -80^◦^C after processing. The sera samples of the selected subjects for this study were taken for the first time from the bio-bank of the SBTCS in Shanghai in May, 2008 for SELDI-TOF-MS. The study protocol was approved by the Institutional Review Boards of all institutes involved in the study. 

### 2.2. Randomized Selection of the Test Sets

Serum samples from 30 cancer cases, 20 cholelithiasis patients, and 20 healthy controls were randomly selected from a total of 110 subjects. Each sample was divided into two 10 μL aliquots with one aliquot used for the training set and the other aliquot used for the duplicate set. For the remaining 40 samples (20 cancer cases, 10 cholelithiasis patients, and 10 healthy controls), one 10 μL aliquot was created for each sample to be included in the blind test set.

### 2.3. Immobilized Metal Affinity Capture (IMAC30) ProteinChip Assay and SELDI-TOF-MS Analysis

Sodium acetate, acetonitrile, trifluoroacetic acid (TFA), sinapinic acid (SPA) and 3-(cyclohexylamino)-1-propanesulfonic acid (CHAPS) were purchased from Sigma-Aldrich (St. Louis, MO, USA). The IMAC30 ProteinChips and PBS IIc SELDI-TOF equipped with an autoloader were purchased from Ciphergen Biosystems Inc. (Fremont, CA, USA).

IMAC30 ProteinChip arrays were prepared by incubation with 100 mM CuSO_4_ for 10 min (50 μL per well) and with 50 mM sodium acetate buffer (pH 4.0) for 2 minutes (10 μL per well). Each incubation was followed by three de-ionized (DI) water rinses. The ProteinChip Assay Cassette was then placed in the Bioprocessor and washed twice for 5 min with 200 mL binding buffer (PBS with 500 mM NaCl, pH 7.2, 200 μL per well). The binding buffer was removed from the wells before adding the sample.

Sera samples were thawed and centrifuged for 10 minutes at 12,000 rpm at 4 °C to exclude insoluble substances, and then were analysed on Cu^2+^-loaded IMAC30 ProteinChip arrays. 6 μL of each sera sample was diluted 40-fold in 234 μL PBS containing 500 mM NaCl (pH7.2), added to each well (200 μL per well), and incubated for 90 min at room temperature on an orbital shaker with shaking at 900 rpm. The ProteinChip arrays were then washed four times using 200 mL of binding buffer (10 min with shaking) followed by a de-ionized water rinse. The ProteinChip arrays were allowed to dry and 0.5 μL of saturated solution of SPA in 50% acetonitrile and 0.15% TFA was applied to each spot. After air drying, another 0.5 μL of SPA was added to each spot and the spots were air-dried again before detection in a PBS IIc SELDI-TOF equipped with an autoloader. Each sera sample was spotted randomly on the surfaces of 25 ProteinChips. Technicians were blinded to the identity of each sample.

TOF spectra were generated by averaging 130 laser shots for each spot with an intensity of 150 µJ and a detector sensitivity of 7, and collected over 0–20 kDa ranges with an optimum range of 3,000–15,000 Da. Spectra were externally calibrated to be within in ±0.1% using NP20 chip with all-in-one standardized protein. Peaks were detected automatically using Ciphergen ProteinChip 3.11 software (valley depth and peak height were set at two times the noise).

### 2.4. Decision tree Classification and Model Assessment

To identify the serum proteins and polypeptides that were significantly different between BTCs and non-cancer sera samples, mass-to-charge ratio (*m/z*) protein peak detection was performed with the Biomarker Wizard module in the mass range from 0 to 20 kDa after normalizing peak intensities to the total ion current. In this study, m/z below 3,000 Da and above 15,000 Da were eliminated from analysis to avoid artifacts caused by the energy absorbing matrix. Therefore, proteins with m/z ranges of 3,000–15,000 Da were included in the analysis for mass calibration, baseline subtraction, and normalization according to total ion current. Valid protein peaks were defined as those with signal to noise ratios (S/N) greater than 5 and with minimum peak threshold (% of all spectra) greater than 10%. The results were exported in .csv format for subsequent analysis. Peaks found to be significantly different between cancers and non-cancers in the training set were used to develop the decision tree classification using the J48 algorithm (java version of c4.5) of R Weka package (http://cran.r-project.org/web/packages/RWeka/index.html). To achieve better results from our decision tree classifier, peaks were ranked by p values from *t*-tests in ascending order and added to the decision tree model one by one until the best performance was achieved. 

### 2.5. Test Set Sample Classification

The validity and accuracy of the optimum decision tree classification were challenged to predict the presence or absence of cancer in sera samples in the duplicate and blind test sets. These samples were arranged randomly and cancer status was unknown to the technicians. Sensitivity and specificity of the decision tree classification were then calculated according to the results.

### 2.6. Assessment of the Reproducibility of SELDI Spectra

The randomly selected 70 sera samples were divided into two aliquots and thus were SELDI-TOF assayed twice. Observation agreement of two assays and kappa value were calculated to evaluate the reliability of the assay. Matched *t*-test was also applied to compare the difference in intensity between two assays among cases and controls. Overall coefficient of variations (CVs) for the five identified *m/z* peaks in 10 replicate assays of one subject were used to further evaluate the reproducibility of SELDI-TOF assay.

### 2.7. Statistical Analysis

Comparisons of parameters between BTCs and controls were performed using two sample *t*-test. *P*-values less than 0.01 were considered statistically significant. Sensitivity was calculated as the number of correctly classified as diseased samples divided by the total number of diseased samples. Specificity was defined as the ratio of the number of negative samples correctly classified to the total number of true negative samples. To assess reproducibility, coefficient of variations (CVs), expressed as a percentage, were calculated as 100 times the standard deviation divided by the mean (CV = 100 × σ/μ) for signal intensity and *m/z* using results from 10 replicate assays from one subject. Small CVs indicate less dispersion from the mean and thus better reproducibility. In order to estimate the performance of decision tree model, permutation test was performed [23]. Since the null hypothesis assumes that the two classes are indistinguishable with respect to the selected statistic, all the training datasets generated through permutations are equally likely to be observed under the null hypothesis, yielding the estimates of the statistic for the empirical distribution. An equivalent result would be obtained if we choose to permute the data, rather than the labels. The p value was calculated by 10,000 permutations.

## 3. Results

### 3.1. Characteristics of Subjects

The selected BTCs and controls were comparable in age and sex, as presented in Table 1. Of 50 cancer cases, eight were stage 0–I, 11 stage II, 12 stage III, 18 stage IV, and one of unknown stage; the difference in distribution between the training and blind test sets was not significant (*P* for χ^2^ test = 0.09). By BTC sub-sites, 19 were gallbladder cancers, eight were extrahepatic bile duct cancers, and three were ampulla of Vater cancers; no significant difference in BTC types was observed between training set and blind test set (*P* for χ^2^ test = 0.99). Within BTC subjects, 66% had gallbladder stones and 26% had stenosis of the bile duct, while in cholelithiasis subjects, all had gallstones but only two were reported with stenosis (data not shown in the table).

**Table 1:** Selected clinicopathologic features of biliary tract cancers (BTCs) and controls.

				Subjects (%)	Males(%)	Females(%)	Age range (years)	Mean age(years)	Training set (%)	Blind set (%)
BTCs				50	15	35	42–75	64.6	30	20
	Tumor site									
		Gallbladder		32 (64.0)	8(53.3)	24(68.6)	43–72	63.9	19(63.3)	13(65.0)
			Extrahepatic bile ducts	13 (26.0)	4(26.7)	9(25.7)	42–74	64.8	8(26.7)	5(25.0)
			Ampulla of Vater	5 (10.0)	3(20.0)	2(5.7)	62–75	68.2	3(10.0)	2(10.0)
									*P* for χ^2^ test = 0.99	
			Clinicopathological stage							
			Stage 0–I	8 (16.0)	4(26.7)	4(11.4)	53–74	65.2	7(23.3)	1(5.0)
			Stage II	11(22.0)	5(33.3)	6(17.1)	43–75	66.3	8(26.7)	3(15.0)
			Stage III	12(24.0)	0(0.0)	12(34.3)	47–70	62.9	7(23.3)	5(25.0)
			Stage IV	18(36.0)	6(40.0)	12(34.3)	42–73	63.9	7(23.3)	11(55.0)
			Unknown	1(2.0)	0(0.0)	1(2.9)	--	74.1	1(3.3)	0 (0.0)
									*P* for χ^2^ test = 0.09	
			Gallstone							
			With	33(66.0)	7(46.7)	26(74.3)	42–74	63.5	20(66.7)	13(65.0)
			Without	17(34.0)	8(53.3)	9(25.7)	43–75	63.5	10(33.3)	7(35.0)
									*P* for χ^2^ test = 0.90	
Cholestasis										
			With	13(26.0)	2(13.3)	11(31.4)	47–74	64.8	11(36.7)	2(10.0)
			Without	37(74.0)	13(86.7)	24(68.6)	42–75	63.7	19(63.3)	18(90.0)
									*P* for χ^2^ test = 0.04	
			Controls	60	21	39	35–74	59.9	40	20
			Cholelithiasis subjects	30(50.0)	10(47.6)	20(51.3)	43–72	58.3	20 (50.0)	10(50.0)
			Healthy subjects	30(50.0)	11(52.4)	19(48.7)	35–74	61.6	20(50.0)	10(50.0)
									*P* for χ^2^ test = 1.00	

### 3.2. SELDI-TOF-MS Data Analysis

**Figure 1:**
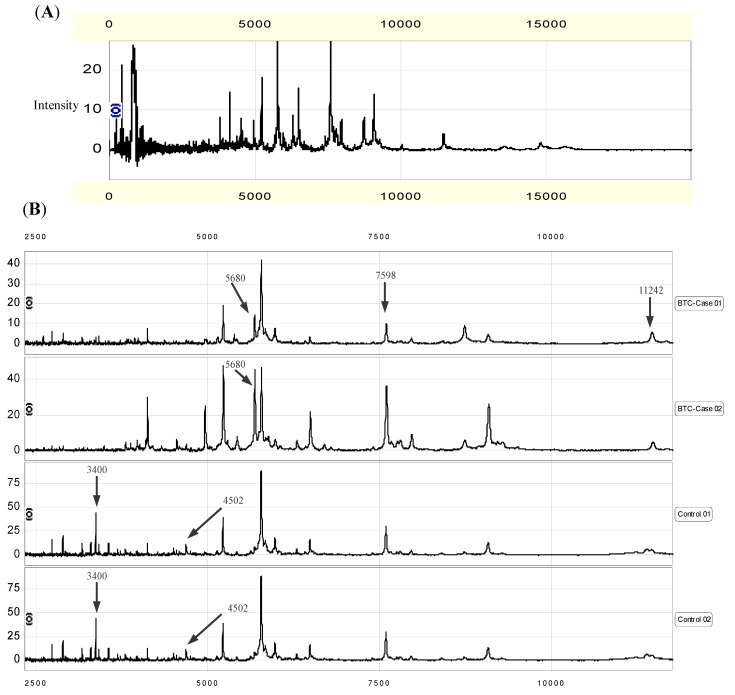
(**A**). Representative protein mass spectra of the sera sample processed on an IMAC30 ProteinChip surface, showing the proteins with masses between 0 and 20,000 *m/z*. (**B**). Detection of five proteins in the mass pattern of serum. Mass spectra of sera samples from two different BTCs (BTC-Case 01 and 02) and two non-cancer controls (Control 01 and 02) were generated on an IMAC30 ProteinChip array. The signal intensity at five proteins was significantly different between the BTCs and the normal specimens. The average molecular masses of the proteins identified as down-regulated in case specimens were *m/z* 3,400, 4,502 and 7,598 Da and as up-regulated were *m/z* 5,680 and 11,242 Da.

Figure 1A is a representative protein spectrum obtained by IMAC30 processing and SELDI-TOF-MS analysis showing the protein masses between *m/z* 0 and 20,000 of a single sera specimen. As shown, the SELDI technology was particularly effective in resolving the molecular weight proteins and polypeptides between *m/z* 3,000 and 15,000 Da.

Of a total of 76 protein peaks identified between *m/z* 3,000 and *m/z* 15,000, no significant differential peaks were observed between cholelithiasis and healthy controls, and the significant peaks between cholelithiasis and BTCs were almost similar to those between the healthy control and BTCs. Therefore, the two non-cancer groups were combined as one control group for further analysis. The signal intensities for 14 proteins were significantly different between the BTCs and the normal specimens (*P* for *t* test < 0.01), with nine down-regulated and five up-regulated in BTCs (Table 2). 

**Table 2:** Significant differential protein peaks between BTCs and controls in the training set.

m/z	P value	Cases (N = 30)			Controls (N = 40)	
		Mean	SD		Mean	SD
2,170	4.23E−05	0.78	1.14		2.87	2.72
2,967	7.93E−07	1.80	2.37		4.93	3.01
3,300	0.0016	1.91	1.38		3.14	1.81
3,400	1.56E−07	1.61	2.49		4.09	2.03
4,188	0.0059	1.55	1.26		3.81	4.64
4,503	2.19E−06	4.01	4.50		9.72	3.57
4,906	8.99E−05	0.07	0.32		0.59	0.84
5,630	0.000969425	1.73	1.55		0.95	0.83
5,681	0.000132676	10.70	9.24		4.46	4.75
7,598	0.0009	23.03	10.82		32.62	10.45
7,797	0.0035	3.54	1.47		4.73	1.53
10,875	0.006116577	0.27	0.35		0.09	0.13
11,167	0.004078681	0.60	0.90		0.13	0.28
11,242	0.002574666	0.89	1.27		0.25	0.41

*Abbreviations*: BTCs = biliary tract cancers; *m/z* = mass-to-charge ratio.

### 3.3. Construction of the Decision Tree Classification

Before constructing the decision tree, all the valid *m/z* peaks in the training (30 cases and 40 controls), duplicate (30 cases and 40 controls) and blind test sets (20 cases and 20 controls) were aligned, and forty peaks between *m/z* 3,000 and *m/z* 15,000 were observed to be valid for the construction of the decision tree classifier. The five most significantly different peaks by intensity levels between BTCs and healthy controls were thus selected as candidate biomarkers to distinguish sera samples of BTCs from those of controls (Table 3); these peaks were named for their molecular weights of 3,400, 4,502, 5,680, 7,598 and 11,242 Da. 

**Table 3:** Significant differential protein peaks used to construct the decision tree classification.

m/z	P value	Cases (N = 80)			Controls (N = 120)	
		Mean	SD		Mean	SD
3,400	0.000000156	1.6057	2.4867		4.0861	2.0254
4,502	0.00000219	4.0145	4.4959		9.7244	3.5676
5,680	0.000132676	10.6997	9.2400		4.4620	4.7509
7,598	0.000890586	23.0274	10.8248		32.6237	10.4506
11,242	0.0025747	0.8937	1.2659		0.2457	0.4131

*Abbreviation*: *m/z* = mass-to-charge ratio.

Of the five peaks, the 3,400, 4,502 and 7,598 Da peptides appeared to be down-regulated, and the 5,680 and 11,242 Da peptide were up-regulated in sera samples from BTCs compared with those from controls, as shown in Figure 1B, in which randomly selected protein mass spectra for two BTC cases and controls were presented as an example. Together, the five peaks appeared to have the best performance. The correct classification rate of 10-fold cross-validation was 78.54%, and the decision tree classifier constructed with the peaks was observed to outperform a pure random guess (*P* for permutation test < 0.0001).

**Figure 2:**
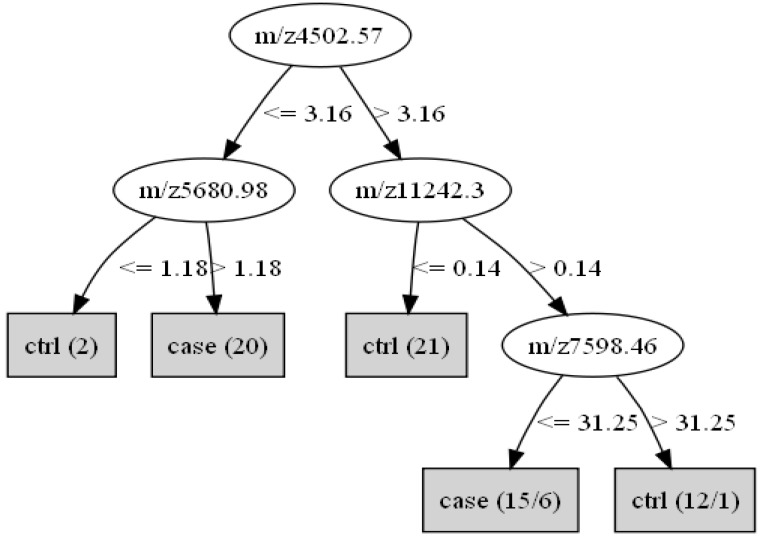
Decision tree classifier of the BTCs and non-cancer groups. The circles show the decision nodes with the peak mass in m/z. Listed besides the arrows are the peak intensity cut-off levels. The three masses form the splitting rules. The final boxes are the terminal nodes classified as being either cancer or normal.

Figure 2 is the constructed decision tree with four (4,502, 5,680, 7,598 and 11,242 Da) of the five selected peaks. The circles in the figure were the decision nodes with the peak mass in *m/z*. Listed besides the arrows were the peak intensity cut-off levels. According to the decision tree, a sera sample with an intensity of more than 3.16 at *m/z* 4,502.57, an intensity of more than 0.14 at *m/z* 11,242.3 and an intensity of 31.25 at *m/z* 7,598.46 would be identified as a control’s sample. As a result, the decision tree achieved a sensitivity of 96.7% (29/30), a specificity of 85.0% (34/40), and a positive predictive value of 82.9% (29 of 35) in diagnosing BTCs in the training set (Table 4). 

### 3.4. Identification of BTC Cases and Controls

As presented in Table 4, the constructed decision tree was able to distinguish BTCs from non-cancers with a sensitivity of 66.7% (20 of 30) and a specificity of 70.0% (28 of 40) in the duplicate test set, and a sensitivity of 95.0% (19 of 20) and a specificity of 75.0% (15 of 20) in the blind test set. Positive predictive values for the duplicate and blind test sets were 62.5% (20 of 32) and 79.2% (19 of 24), respectively. 

**Table 4:** Results from the decision tree algorithm in the training, duplicate and blind test sets.

Data set		Identified as BTCs			Identified as normal	
		N	%		N	%
Training set						
	BTCs (N = 30)	29	96.7 ^a^		1	3.3
	Normal (N = 40)	6	15.0		34	85.0 ^b^
Duplicate test set						
	BTCs (N = 30)	20	66.7 ^a^		10	33.3
	Normal (N = 40)	12	30.0		28	70.0 ^b^
Blind test set						
	BTCs (N = 20)	19	95.0 ^a^		1	5.0
	Normal (N = 20)	5	25.0		15	75.0 ^b^

^a^ Sensitivity and ^b^ specificity in the training, duplication and blind test sets. *Abbreviation*: BTCs = biliary tract cancers.

We further checked the sensitivity and specificity of the decision tree according to the site and clinicopathological stage of BTCs, as well as the presence of cholestasis in cancers, and did not find any significant difference in these subgroups (data not shown). Particularly, five of eight early stage BTCs were correctly identified as cases, which was not significantly different from that for advanced staged cancers (*P* = 0.08 for Fisher’s exact test). Considering that patients with cholangiocarcinoma in the extrahepatic bile ducts are more likely to have jaundice, we also compared the sensitivity and specificity between the BTCs with and without obstructive jaundice, but did not observe a significant difference (data not shown). Additionally, we found that the decision tree correctly identified the two cholelithiasis controls with cholestasis in this study as controls.

### 3.5. Reproducibility of the Assays

As shown in Table 5, the observed agreement of classification results according to the decision tree between the training set and the duplicate set was 71.4%, with a kappa value of 0.43 (u = 3.98, P < 0.01). Further analysis showed that the difference in intensity between two assays was not significant for the 3,400, 5,680, 7,598, and 11,242 Da peptides but reached significance for the 4502 Da peptide among both cases (*P* for matched *t*-test = 0.0071) and controls (*P* for matched *t*-test = 0.03) (Table 6). 

**Table 5:** Agreement of classification results according to the decision tree between the training set and duplicate set.

Duplicate test set	Training set		Total
	BTCs	Normal	
BTCs	23	8	31
Normal	12	27	39
Total	35	35	70

Observation agreement = 71.4%; Kappa = 0.43, u = 3.98, P < 0.01; *Abbreviation*: BTCs = biliary tract cancers.

**Table 6:** SELDI-TOF-MS assay reproducibility of twice assays of 70 sera samples.

Protein peak(Da)	All subjects (n = 70)			BTCs (n = 30)			Controls (n = 40)		
	Difference in intensity (mean ± SD)	*P for matched* *t-test*		Difference in intensity(mean ± SD)	*P for matched* *t-test*		Difference in intensity (mean ± SD)	*P for* *Matched* *t-test*	
3400	−0.2635 ± 3.0660	*0.47*		−0.1313 ± 2.4212	*0.77*		−0.3626 ± 3.4998		*0.52*
4502	−0.1007 ± 4.0941	*0.84*		−1.9796 ± 3.7785	*0.0076*		1.3084 ± 3.7798		*0.03*
5680	0.3937 ± 3.2844	*0.32*		0.2291 ± 4.0356	*0.76*		0.5172 ± 2.6040		*0.22*
7598	0.8956 ± 8.6985	*0.39*		1.6750 ± 6.7575	*0.19*		0.3110 ± 9.9545		*0.84*
11242	−0.075 ± 0.5852	*0.29*		−0.1597 ± 0.8023	*0.28*		−0.0118 ± 0.3428		*0.83*

*Abbreviations*: SELDI-TOF-MS = surface-enhanced laser desorption/ionization time-of-flight mass spectrometry; BTCs = biliary tract cancers.

We further calculated the overall percentage CVs for the five identified m/z peaks in 10 replicate assays of one sample. The mean masses, SDs, and overall CVs for detected protein peaks are shown in Table 7. The overall CVs for intensity were 68.8% for *m/z* 3,400, 61.0% for *m/z* 4,502, 32.7% for *m/z* 5,680, 15.8% for *m/z* 7,600, and 39.0% for *m/z* 11,242 Da. The CVs for *m/z* ranged from 0.0% to 0.05%. The data showed that the reproducibility of protein detection using SELDI-TOF-MS was not satisfactory.

**Table 7:** SELDI-TOF-MS assay reproducibility of 10 times duplicate assays of one sera sample.

Protein peaks (Da, mean ± SD)	CV for Intensity (%)	CV for m/z (%)
3,400.04	68.8	0.00
4,502.57	61.0	0.00
5,685.76±3.08	32.7	0.05
7,600.15±3.40	15.8	0.04
11,242.30	39.0	0.00

*Abbreviations*: SELDI-TOF-MS = surface-enhanced laser desorption/ionization time-of-flight mass spectrometry; BTCs = biliary tract cancers; CV = coefficient of variation.

## 4. Discussion

Since Petricoin *et al*. [12] have reported identification of a serum protein cluster pattern that segregated ovarian cancers from non-cancers with high sensitivity and specificity, protein profiles have been developed through SELDI-TOF-MS for early detection or as discriminate diagnostic tools for a growing list of cancers, particularly for those with poor survival such as that of the ovaries [12], lung [11], liver [10] and pancreas [13]. While evidence supporting the use of SELDI profiling technique as a potential tool for cancer diagnosis is rapidly accumulating, concern about the reproducibility of the methods is also growing [24,25].

To date, only two studies have identified specific protein profiles for intrahepatic cholangiocarcinoma [14,15]. Scarlett *et al.* [14] compared the serum profiles of 20 cholangiocarcinoma with those of 20 benign diseases and 25 healthy volunteers and found that the *m/z* 4,462 peak had superior discriminatory ability compared to the currently used biomarkers, CA19-9 and CEA; the training models developed with panels of peaks from serum protein profiles could distinguish patients with cholangiocarcinoma from those with benign lesions with 65.0% sensitivity and 70.0% specificity, and from sera of healthy volunteers with 75.0% sensitivity and 100% specificity. Liu *et al.* [15] analyzed 427 serum samples including 56 cholangiocarcinomas, 49 hepatobiliary diseases, 269 other cancer controls and 53 healthy individuals using SELDI technology and discovered three biomarker panels (*m/z* 13.76, 13.88, and 14.04 kDa) that could effectively distinguish cholangiocarcinoma from benign biliary diseases and normal individuals. However, these biomarkers were not tested on BTCs.

In the current study, we used the SELDI-TOF-MS in combination with the J48 algorithm of the R Weka package to profile protein signatures of sera samples from BTCs cases and controls. Five peaks corresponding to 3,400, 4,502, 5,680, 7,598, and 11,242 Da peptides were identified as top differential proteins for BTCs and used to construct a decision tree classification with a sensitivity and a specificity as high as those previously constructed for intrahepatic cholangiocarcinoma [14,15]. The sensitivity and specificity appear higher than those reported for CA19-9 and CEA, the main serum markers currently used in diagnosis of BTCs [26,27,28]. Our results suggest diagnostic potential of the SELDI technique in BTCs.

In this study, we found that the sensitivity and specificity of the constructed decision tree in detecting cases were not significantly different between the BTCs at different clinicopathological stages. These results indicate a promising role of the profile as an early detection biomarker for BTCs. Another important finding in this study is that the BTCs, regardless of the tumor site, shared a similar protein profile. The common embryologic origin of gallbladder and bile ducts may explain the findings. However, we could not exclude the possibility that the proteomic changes may not be cancer specific but may be due to presence of cholestasis, a symptom usually occurred in BTCs. In this study, however, the sensitivity and specificity were not significantly different between the BTCs with and without obstructive jaundice, and the only two cholelithiasis controls with cholestasis in this study were correctly identified as the normal, both of these findings help to partly alleviate our concern. 

It is of note that the sensitivity and specificity were much lower in the duplicate set than in the training set, suggesting unsatisfactory reliability of the SELDI technique. Therefore, we further evaluated the reproducibility of the technique through two approaches. In the first approach, we compared the intensity of specific peaks in the training set and in duplicate testing set as well as to calculate agreement and Kappa value of the two assays. For the second approach, we assayed one sample 10 times and calculated the overall CVs for intensity and *m/z* of differential peaks. The results from both approaches suggest that the reproducibility of protein detection using SELDI-TOF-MS was not satisfactory. The low assay reproducibility may limit the application of the technique in clinical practice. Therefore, how to improve the assay reproducibility is vital for the extensive application of the technique. Dividing target proteins using different methods or inonizing proteins in double charge instead of single charge may be help to achieve the goal. 

The strengths of this study include the representative samples of BTCs, cholelithiasis and healthy controls, multiple comparisons of the subjects, and comprehensive evaluation of reproducibility of SELDI technique. Due to the limited number of samples (n = 110) in this analysis, however, our results need to be considered as preliminary. In this study, we did not assay the CA19.9 and CEA, thus we could not evaluate the combined sensitivity and specificity of these biomarkers with the protein profiles. Moreover, the sera samples used in this study had been stored at −80 °C for a few years. The long term storage of the samples may lead to degradation of certain proteins. However, this study was the first thaw of the sera samples and thus effects of freeze-thaw cycles are minimized. In addition, we could not compare the differential protein peaks between malignant and benign lesions since subjects with benign disease were not included in the parent study. We also did not isolate, identify and quantify the differential proteins, which limited our ability to understand the mechanisms underlying the difference in protein expression between BTCs and non-cancers. 

## 5. Conclusion

In summary, our pilot study suggests that serum protein profiling by SELDI-TOF MS analysis can distinguish BTCs from non-cancers with higher sensitivity and specificity than other non-invasive methods. Therefore, although the low reproducibility raises a question on the validity of the technique, SELDI-TOF MS is meaningful as a diagnostic tool for BTCs. Reproducibility of the technique would need to be improved in order to extend its application in clinical practice. 
